# Use of Photobioreactors in Regenerative Life Support Systems for Human Space Exploration

**DOI:** 10.3389/fmicb.2021.699525

**Published:** 2021-06-29

**Authors:** Jana Fahrion, Felice Mastroleo, Claude-Gilles Dussap, Natalie Leys

**Affiliations:** ^1^Interdisciplinary Biosciences Group, Belgian Nuclear Research Centre (SCK CEN), Mol, Belgium; ^2^CNRS, SIGMA Clermont, Institut Pascal, Université Clermont Auvergne, Clermont-Ferrand, France

**Keywords:** space exploration, bioregenerative life support systems, microalgae, photobioreactors, air revitalization, cyanobacteria

## Abstract

There are still many challenges to overcome for human space exploration beyond low Earth orbit (LEO) (e.g., to the Moon) and for long-term missions (e.g., to Mars). One of the biggest problems is the reliable air, water and food supply for the crew. Bioregenerative life support systems (BLSS) aim to overcome these challenges using bioreactors for waste treatment, air and water revitalization as well as food production. In this review we focus on the microbial photosynthetic bioprocess and photobioreactors in space, which allow removal of toxic carbon dioxide (CO_2_) and production of oxygen (O_2_) and edible biomass. This paper gives an overview of the conducted space experiments in LEO with photobioreactors and the precursor work (on ground and in space) for BLSS projects over the last 30 years. We discuss the different hardware approaches as well as the organisms tested for these bioreactors. Even though a lot of experiments showed successful biological air revitalization on ground, the transfer to the space environment is far from trivial. For example, gas-liquid transfer phenomena are different under microgravity conditions which inevitably can affect the cultivation process and the oxygen production. In this review, we also highlight the missing expertise in this research field to pave the way for future space photobioreactor development and we point to future experiments needed to master the challenge of a fully functional BLSS.

## Introduction

Human space exploration aims to go farther into space and crewed missions are planned to Moon and Mars. The European Space Agency (ESA) as well as the National Aeronautics and Space Administration (NASA) plan human missions to Mars in the coming decades (Hufenbach et al., 2014; Anderson et al., 2019). The space travelers need oxygen, fresh water and nutritional food to survive on such space missions (MacElroy and Bredt, 1984) and the supply has to become independent from Earth. In order to minimize the resupply needs and the embarked mass, the recycling process will have to include food production coupled to oxygen (O_2_) and water (H_2_O) recovery that entails the use of at least one biological compartment for producing edible biomass. This challenge can be solved by the development of a bioregenerative life support system (BLSS), that meets the needs at least for a part of food supply to the crew and secures air, water as well as safe waste recycling (Gitelson et al., 1976).

This review will focus on the bioprocess of microbial photosynthesis and, in particular, on photobioreactors (PBRs) used in space for BLSS. Moreover, we focus on the process of air revitalization, meaning the efficient removal of carbon dioxide (CO_2_) and production of O_2_. The goal of this study is to give an overview of the experiments that have been conducted with liquid cultures of photosynthetic microbes and PBRs for application in space, and to highlight similarities and common challenges. Hereby, the last 30 years will be focused. Additionally, missing data and suggestions on future experiments will be discussed. The following sections describe the general requirements for life support systems and the current state of the art to how a BLSS can be developed using different techniques and organisms.

## Oxygen Requirements and Carbon Dioxide Limits in Space Habitats

In our daily lives, O_2_ is freely available to us because our atmosphere functions as an infinite buffer tank for O_2_ produced by Earth’s photosynthetic biosphere. However, when humanity wants to explore space, closed spacecraft do not have an endless supply of O_2_. Due to the human respiration process, the O_2_ level decreases while CO_2_ and water vapor (H_2_O_vapor_) increase inside a closed habitat over time. The typical respiratory rate for a healthy adult at rest is 12–18 breaths per minute or roughly 20,000 breaths a day (Crockett et al., 2018). In spacecraft environments, it is considered that one standard 82 kg crew member consumes 0.82 kg d^–1^ O_2_ and produces 1.04 kg d^–1^ CO_2_ and 1.85 kg d^–1^ H_2_O_vapor_ during intravehicular activities. Based on these values, the respiratory quotient (mole CO_2_ produced per mole O_2_ consumed) is 0.92, but the respiratory quotient varies depending on the physical workload, diet and individual metabolism (Anderson et al., 2018). When the partial pressure of CO_2_ in the air becomes too high, it becomes toxic to humans. For example, a maximum value for CO_2_ of ≤0.52 kPa (0.52% per volume or 5,200 ppm) is allowed on the International Space Station (ISS) (Anderson et al., 2018) and even 0.50% (5,000 ppm) CO_2_ in a habitat is thought to have a negative impact to human health over longer periods. In order to keep the risk of headache development under 1%, the average value over 7 days should not exceed ∼0.33% CO_2_ (Law et al., 2014). Consequently, a system is needed to remove excess CO_2_ and provide a sufficient partial O_2_ pressure. The partial O_2_ pressure depends on the total cabin pressure and should range between 18 and 23.1 kPa or 21–50% per volume, respectively (Lange et al., 2003; Swickrath and Anderson, 2012; Anderson et al., 2018). On the ISS, the total pressure is set at 101.3 kPa (with 21% O_2_), so it resembles the pressure on Earth (101.325 kPa on sea level). But other pressure regimes are also suitable for manned habitats. When using atmospheres consisting of mixed gasses (e.g., N_2_/O_2_ mixtures), total pressures between 48.0 and 102.7 kPa are acceptable. In early space missions, almost pure O_2_ atmospheres at total pressure of 34.5 kPa were used. Due to the increased fire hazard under pure oxygen atmospheres, nowadays spacecraft use gas mixtures similar to our atmosphere (Lange et al., 2003; Anderson et al., 2018).

## Physicochemical Air Revitalization in Space Habitats

To date all crewed spacecraft solely rely on physicochemical methods of air revitalization. In the first years of crewed spaceflight, different physicochemical methods were tested. Early space missions like Mercury, Gemini, the Apollo Command Module and the Apollo Lunar Module used lithium hydroxide (LiOH) canisters that remove CO_2_ by converting atmospheric CO_2_ to lithium carbonate (Li_2_CO_3_) (Winton et al., 2016). The canisters have to be replaced after usage because the chemical reaction is irreversible. Additionally, different molecular sieves were used to remove CO_2_ and H_2_O_vapor_. O_2_ storage was usually accomplished via high pressure liquid O_2_ or chemically bound O_2_.

On the ISS, a Carbon Dioxide Removal Assembly (CDRA), an Oxygen Generation Assembly (OGA) and a Carbon Dioxide Reduction Assembly (CRA), are currently used to maintain a suitable gas balance. These systems use regenerable absorbent materials (in CDRA), water electrolysis (Eq. 1) (in OGA) and the Sabatier reaction (Eq. 2) (in CRA) to remove CO_2_ and produce potable water (H_2_O_potable_) (Mansell et al., 2011; Knox and Stanley, 2015; Takada et al., 2019).

(1) 2HO2→2H+2O2

(2) CO+24H→2CH+42HO2

The methane produced in the CRA is vented into space. Considering a respiratory coefficient near than unity, the mol quantities of O_2_ to produce and CO_2_ to remove are almost equal. This means that the stoichiometric eqs. (1, 2) show that there is a global hydrogen imbalance. Either it must be compensated by hydrogen resupply or excess CO_2_ has to be removed by absorption to materials that end up as trash. As a consequence, substantial amounts of carbon and other elements are lost over time (Mansell et al., 2011; Knox and Stanley, 2015). So even nowadays, all of the methods used for air revitalization are physicochemical and not regenerative (Junaedi et al., 2011; Tobias et al., 2011). They use a lot of consumables and produce a lot of waste and are therefore only applicable for near-by (LEO) missions with easy resupply from Earth (Daues, 2006).

## Bioregenerative Air Revitalization in Space Habitats

Possible solutions to this problem are photosynthetic biological systems using plants, algae and cyanobacteria for air revitalization and carbon recycling coupled to production of edible biomass. The photosynthetic activity of these organisms captures CO_2_ and H_2_O to produce edible biomass and O_2_. The development of a BLSS to ensure the autonomous air supply of space crews on distant and long-duration missions, became a long time goal of many space agencies (Lasseur et al., 2010). The first ideas on this topic were already developed around 1900 (Salisbury et al., 1997) and the development process has already started in the 1960s before the first human flew to space. Many different attempts to obtain such a BLSS with efficient biological air revitalization were conducted during the second half of the 20th century. Especially the soviet space program investigated different approaches using microalgae and higher plants very early (Hooke et al., 1986; Niederwieser et al., 2018).

Additionally, several test sites were developed on Earth. Some examples of large ground based studies are the BIOS-I project, where the algae *Chlorella* provided O_2_ for one human and the BIOS-III project that inhabited three crew members; plants and green algae performed at the BIOS facility in Krasnoyarsk, Siberia, Russia (Kirensky et al., 1968; Gitelson et al., 1976; Gitelson, 1992). The NASA Biomass Production Chamber project was a closed greenhouse designed to grow crops on a small area (20 m^2^), that was successfully operated for over 1,200 days (Wheeler et al., 1996). Another example is the Biosphere-2 project (in Oracle, AZ, United States), which tried to mimic the terrestrial biosphere by inhabiting eight crew members together with various plants (Allen and Nelson, 1997). A recent example is Lunar Palace 1, that investigated a system consisting of plants, insects and three crew members for 105 days (Fu et al., 2016). ESA has initiated the development of its own Micro-Ecological Life Support System in 1988, called MELiSSA, combining physicochemical technologies with microbial and plant conversion of organic and inorganic waste into nutrients, and biological air revitalization (Lasseur et al., 2010). In addition to carbon, hydrogen and O_2_ balances, the MELiSSA loop considers the recycling of nitrogen, which in turns incorporates food production by biological compartments coupled to the other balances. The MELiSSA loop consists of five interconnected compartments including two PBRs. Compartment III holds a nitrifying culture to produce nitrate for compartments IVa (cyanobacteria) and IVb (higher plants), which use the nitrate, excess CO_2_ from the crew and light to produce edible biomass, potable water and O_2_ (Gòdia et al., 2002). The photosynthetic cyanobacterium *Limnospira indica*, formerly known as *Arthrospira* sp. PCC8005 (Nowicka-Krawczyk et al., 2019) is used as part of the O_2_ and edible biomass production in one of the PBRs (Poughon et al., 2020).

## Photosynthetic Microorganisms as Catalysers for Air Revitalization in Space

As mentioned above, air revitalization can be achieved by photoautotrophic organisms like cyanobacteria, algae or plants. In general, algae and cyanobacteria have many benefits compared to land plants for CO_2_ removal, and O_2_ and biomass production. Due to these benefits, unicellular photosynthetic organisms are investigated as key component of life support systems for a long time (Averner et al., 1984; Niederwieser et al., 2018).

### Oxygen Production and Carbon Fixation

The light intensity is a key factor for O_2_ production in photosynthetic organisms. On the surface of Earth, the light intensity has a maximum of 2,500 μE m^–2^ s^–1^ (full sunlight, μE = μmol photons) and on average, a daily dosage of 60 mol photons m^–2^ is reached (Melis et al., 1998). Cyanobacteria like *Limnospira* spp. are very potent O_2_ producers. For example, ponds in southeastern California release about 16.8 t of O_2_ ha^–1^ yr^–1^ while fixating 6.9 t of CO_2_ ha^–1^ yr^–1^. In comparison, trees produce about 2.5–11 t of O_2_ ha^–1^ yr^–1^ and fixate 1–4 t of CO_2_ ha^–1^ yr^–1^ (Henrikson, 2010). It has been found, that *Limnospira* cultures are about 2.5 times more productive in tropical environments and under these warm and humid conditions, a pond surface of approximately 80 m^2^ would meet the oxygen needs of one crew member. It was stated that the use of modern photobioreactors that e.g., control temperature, humidity, CO_2_ influx and illumination could reduce the needed surface significantly (Verseux et al., 2015).

In BIOS-I, the experimental data proposed that 20 L or respectively, an illuminated surface of 8 m^2^ of *Chlorella* culture is needed to supply one crew member. In comparison, the BIOS-3 experiment investigated how big a plant compartment has to be to sufficiently provide oxygen for one human and found that the surface would need to be 30 m^2^ (Gitelson, 1992). Javanmardian and Palsson (1992) showed that a 200 L PBR (calculated from a 600 mL prototype) containing *Chlorella vulgaris* is able to meet the gas exchange needs of one human and they proposed that the system could be downsized to a 20 L *Chlorella vulgaris* tank reactor if equipped with an optimized illumination system and regime. In addition, it was shown and experimentally demonstrated that the O_2_ production and CO_2_ consumption of a 83 L PBR with *Limnospira indica* is adaptable via the light intensity and can meet the needs of a three rat compartment (∼5–10% of the O_2_ requirements of one human) in the MELiSSA Pilot Plant facility at the Universitat Autònoma de Barcelona. In this experiment, the rats were kept alive for several months through gas exchange with the PBR (Alemany et al., 2019).

### Biomass Production

Algae and cyanobacteria are also more efficient for edible biomass production in many cases, as they need less surface or volume for biomass production than higher plants, can be continuously harvested, and are fully comestible without complex food preparation and with reduced waste production. Wheeler et al. (2003) presented biomass productivity data (edible dry weight biomass per area and day) of different plants and showed that wheat plants are able to produce up to 12.6 g m^–2^ d^–1^ and soybeans up to 6 g m ^–2^ d^–1^. In comparison, in open ponds, *Limnospira indica* yields up to 15 g m ^–2^ d^–1^ (dry weight) (Jimenez et al., 2003) and *Spirulina* sp. LEB-18 can yield up to 69 g m ^–2^ d^–1^ (dry weight) (Morais et al., 2009). Helisch et al. (2020) also reported high biomass production rates (1.3 g L^–1^ d^–1^ (dry weight per volume and day) for *Chlorella vulgaris* in a microgravity capable PBR (see Table 1, light intensity: 200–300 μE m^–2^ s^–1^). In addition, Zhang et al. (2018) observed generation times between 13 and 28 h for several cyanobacterial species at a light intensity of 30 μE m^–2^ s^–1^. Therefore, harvesting can be done continuously or several times a week, even under low light intensities. On the other hand, typical staple crop plants like rice, wheat or potato, usually take several months until they are harvestable (Watson et al., 2018). In fact, there are large discrepancies between the reported biomass volumetric productivities values. This is due to the fact that microalgae cultures are generally limited by light energy supply so that the production rate depends on the illuminated surface and the light intensity. Therefore, the productivities primarily depend on culture design (illuminated surface versus culture volume) and operational variable (light energy flux). A departure value of light energy yield by photosynthetic systems is in the order of magnitude of 20 mol of photons per mol of carbon fixed (Cornet and Dussap, 2009; Poughon et al., 2020). This is consistent with the maximum value of 69 g m ^–2^ d^–1^ reported by Morais et al. (2009) with a maximum possible light intensity before the appearance of photo inhibition.

**TABLE 1:** PBR ground experiments with the eukaryotic algae *Chlorella vulgaris*.

Hardware	Gas exchange	Volume	Light intensity	Mode	Duration	Results	Authors
PBR	Hollow fiber cartridges	600 mL	0.6 mW/cm^2^ (usable light) ≈ 27.6 μE m^–2^ s^–1^	Batch and continuous	>2 months	The measured oxygen production rate under continuous operation (4–6 mmol/L*h) meets the expectations	Javanmardian and Palsson (1992)
PBR with a vertical rectangular slab-shaped illumination chamber	Hollow fiber cartridges	70–340 mL, depending on the experiment and the number of illumination chambers	25 mW/cm^2^ (on each LED plate) ≈ 1,150 μE m^–2^ s^–1^ via LED	Continuous	10 days	The general performance of the hollow fiber PBR was comparable to the PBR using a sparging system, but the oxygen production rate was decreased	Lee and Palsson (1995)
Plate PBR with automated control system	Unknown	1.5 L	2 LED panels with 117–143 μE m^–2^ s^–1^	Continuous	6 months	A completely closed water cycle could be achieved in the biological system containing multiple organisms. A sufficient gas exchange was also achieved.	Tong et al. (2011, 2012)
Plate PBR with automated control system	Unknown	1.5 L	150, 300, and 350 μE m^–2^ s^–1^	Continuous	192 days	*C. vulgaris* can be used as an emergency system in case of high plant system problems. Additionally, the CO_2_ and O_2_ concentrations could be kept in a good range.	Li et al. (2013)
Raceway PBR	Microgravity-capable membrane	650 mL	200–300 μE m^–2^ s^–1^	Repeated batch mode	188 days	Achievement of biomass growth up to a maximum of 12.2 g/l. The bioreactor works on Earth and is ready to be tested in Space (PBR@LSR)	Helisch et al. (2020)

### Use of Biomass as Food Supplement

Also, the full biomass of several microalgae is edible producing no waste like non-edible roots, stems or leaves. For example, the algae *Chlorella vulgaris* is rich in protein (up to 58%) and contains all essential amino acids (Becker, 2007). It is also rich in unsaturated fatty acids, carotenoids, dietary fibers, vitamins, and minerals (Safi et al., 2014). The cyanobacterium *Limnospira* is also known for its great nutritive value including proteins of high quality, many minerals, vitamins and phytopigments (Farag et al., 2015). It was shown by Morist et al. (2001) that *Limnospira indica* can be used to produce healthy and nutritious food using different methods like freeze-drying, spray-drying and pasteurization. In addition, the consumption of *Limnospira* has beneficial features for space travelers because of its antioxidant and anti-inflammatory treats (Wu et al., 2016). Furthermore, it was shown that *Limnospira indica* is highly irradiation resistant (survival up to at least 6400 Gy of gamma rays) and therefore no negative effects from the increased ionizing irradiation in space are to be expected (Badri et al., 2015).

As algae and cyanobacteria can be cultivated axenic or defined xenic in bioreactors, they do not contribute possibly harmful microorganisms to the microbiome of a spacecraft (Helisch et al., 2020). In comparison, several crops need parts of their own microbiome for optimal growth and contain a risk of catching molds, when grown in spacecraft simulations (Saleem et al., 2019; Fahrion et al., 2020).

Despite the many advantages, using cyanobacteria and algae also has some disadvantages. For instance, cyanobacteria and algae contain less fiber then the edible biomass of plants and too few carbohydrates for a complete diet (Lehto et al., 2006). The nucleic acid content is too high in some microorganisms and some are also too high in certain minerals, which can have a negative effect on the health of the crew members (Averner et al., 1984). Lastly, nutrition from algae and cyanobacteria alone also has a negative impact on certain social aspects. Because photosynthetic microorganisms are very nutrient dense, only a few grams are usually ingested. Preparing, consuming and sharing food is impaired in this case, however, it was shown that the social aspect of eating is important for our mental and physical well-being (Chappuis et al., 2020). Furthermore, being around plants has a positive effect on the mental wellbeing of humans (Lehto et al., 2006). Ideally, a system connecting both should be aimed for as combining a cyanobacterial or algae PBR with a higher plant compartment increases the variety of the consumed food and was also shown to increase the recycling rates of a BLSS, and will also provide system redundancy for the essential CO_2_ removal and O_2_ production for the crew members (Gros et al., 2003).

## Development of Biological Air Revitalization Systems for Space

The development of biological air revitalization in space is a tedious process. Even before a bioreactor is functional, several key aspects need to be addressed. One of the first tests is to investigate whether the target organism is able to survive a space upload, exposure and return. Different space exposure experiments were launched to address questions of survivability and resistance mechanisms of terrestrial microorganisms to microgravity and ionizing irradiation (Sancho et al., 2007; Onofri et al., 2012; de Vera et al., 2019). Additionally, the organisms are investigated for their growth kinetics, metabolic pathways and genetic stability, in engineered and space cultivation conditions. These primal experiments usually use liquid batch cultures. In a next step, a suitable kinetic model for all important parameters (e.g., in the case of cyanobacteria: O_2_ production, CO_2_ uptake, biomass production, use of nutrients, etc.) has to be developed and tested (Poughon et al., 2020). Only if the organism suits all requirements, experiments with fed-batch or continuous liquid cultures can start. For these tests, suitable PBR hardware has to be created. Sterility, biocompatibility, sealing and proper mixing are some of the requirements here. In addition, sufficient pumps for nutrient delivery and removal of biomass and products are needed (Ai et al., 2008). Depending on the organism, different pH, temperature and nutrient control systems have to be installed. For phototrophic organisms, adequate illumination and gas transfer is necessary. Typically, specific membranes are used frequently to improve the transfer of gasses like CO_2_ and O_2_ to and from the liquid phase (Sarbatly and Suali, 2013).

As soon as a bioreactor and its control system are set up, the interconnection to other compartments of the BLSS has to follow.

### Development of Space PBRs on Earth

Most of the research for biological air revitalization is executed on Earth. Tables 1–3 give an overview of the different ground experiments that were performed in the last 30 years for the development of PBRs for O_2_ production with *Chlorella vulgaris, Limnospira indica* and microalgae in combination with nitrifying communities providing the necessary nitrogen source for algal growth. The hardware and conditions that were used and the corresponding achievements are shown. The volumes for the experiments with *Chlorella vulgaris* were in lab scale and range between 70 mL and 1.5 L. Different PBR shapes were used, and several interesting discoveries were made. Javanmardian and Palsson (1992) showed that the calculated O_2_ production and growth rate fits the experimental value and Li et al. (2013) concluded that a *Chlorella vulgaris* PBR can be used as an emergency O_2_ production system, in case of failure of a plant unit. Four of the five experiments shown in Table 1 persisted for over 2 months. Therefore, longer durations were covered, especially when compared to the experiments in Table 2. The ground experiments with *Limnospira indica* in continuous mode were mainly conducted in cylindrical PBR. With exception of the experiment described in Farges et al. (2009), only the batch cultures were grown in rectangular flasks or Erlenmeyers. Models and their application to the set up were investigated in several experiments. For example, Alemany et al. (2019) validated a model for a two-compartment system and Cornet and Dussap (2009) tested the functionality of different hardware setups and defined a model to predict the O_2_ and biomass productivities of *Limnospira indica*. Table 3 displays three experiments using an interconnected system of nitrifying compartments to microalgae. It is an important step in the development of a functional BLSS to use nitrified urine as nitrogen source for algae and cyanobacteria. In an already quite sophisticated approach, Gòdia et al. (2002) showed the successful combination of a nitrifying bioreactor with a PBR containing *Limnospira indica*. The two other experiments showed that microalgae can be grown successfully on different nitrogen sources.

**TABLE 2:** PBR ground experiments with *Limnospira indica.*

Hardware	Gas exchange	Volume	Light intensity	Mode	Duration	Results	Authors
Batches: rectangular PBR, cont.: cylindrical PBR	Airlift	Batches: 1 L and 4 L, cont.: 7 L	Batch: via white fluorescent lamps (20 W), continuous: via halogen lamps (20 W)	Batch and continuous	Unknown	A model to couple radiant light transfer and growth kinetics is proposed	Cornet et al. (1995)
PBR, 2 identical cylinders	External loop airlift	77 L	Batch: 95 W/m^2^ (≈ 437 μE m^–2^ s^–1^), cont.: 133 W/m^2^ (≈ 611.8 μE m^–2^ s^–1^) via white halogen lamps (20 W)	Batch and continuous	400 h (16.66 days)	Scaling up a 7 L to 77 L PBR was successful and the developed model was applicable	Vernerey et al. (2001)
PBR, 2 identical cylinders	External loop airlift	77 L	Incident light flux 133 W/m^2^ (≈ 611.8 μE m^–2^ s^–1^) via white halogen lamps (20 W)	Continuous	Unknown	Three food preparation methods can be used to process *L. indica* to constitute a safe food source	Morist et al. (2001)
Cylindrical PBR	Airlift	5 L	Batch: 88 W/m^2^ (≈ 404.8 μE m^–2^ s^–1^), continuous: 122 W/m^2^ (≈ 561.2 μE m^–2^ s^–^1) and 150 W/m^2^ (≈ 690 μE m^–2^ s^–1^) via white halogen lamps (20 W)	Batch and continuous	Batch: ∼14 days, continuous: ∼50 days	The nutrient uptake rates of Zn, Fe, Mn, Mg, Cu, and K by *L. indica* have been successfully characterized	Cogne et al. (2003a)
Cylindrical PBR	Airlift	5 L	20–230 W/m^2^ (≈ 92–1,058 μE m^–2^ s^–1^)	Continuous	Unknown	The metabolic network of *L. indica* was characterized and revealed some interesting constraints	Cogne et al. (2003b)
Cylindrical PBR	Mixing and headspace	132 mL	Via halogen lamp	Batch	Up to 400 h	*L. indica* could be grown in a newly designed PBR. Pressure, pH, temperature and cell density were monitored online	Cogne et al. (2005)
One-cylindrical PBR	Hollow fiber membrane	31.8 L (calculated from measures in the reference)	300 μE m^–2^ s^–1^	Continuous	7 days	Successful operation of the bioreactor and suitable control mechanisms could be demonstrated	Ai et al. (2008)
Rectangular PBR	Headspace with tubing and Peltier condensers, aeration via tube	600 mL (batch)	Via red and blue LEDs, two different LED panels	Batch and continuous	Up to 3.500 h	Red LEDs cause the same biomass productivity in a PBR with *L. indica*, but with a much lower energy consumption	Farges et al. (2009)
Eight different PBRs, different volumes, shapes, illumination	Depending on the PBR, mostly airlift	0.1–77 L	Photon fluxes between 30 and 1,600 μE m^–2^ s^–1^	Batch and continuous	Cont. cultures: at least six residence times	Successful presentation of an analytical formula to predict the productivities of *L. indica* in different PBRs	Cornet and Dussap (2009)
Erlenmeyer flasks (batch), cylindrical PBR	Unknown	250 mL and 2 L	43 μE m^–2^ s^–1^ (batch), 140 μE m^–2^ s^–1^ (PBR)	Batch and continuous	Up to 50 days (PBR)	Urea seems to be a better nitrogen source than NH_4_^+^. Pulse feeding might help to avoid inhibitory effects	Deschoenmaeker et al. (2017)
Cylindrical double jacketed PBR	Purging with N_2_, stirring with turbine	2 L	60 μE m^–2^ s^–1^ (batch) and 300 ± 50 μE m^–2^ s^–1^ (PBR), radially illuminated	Batch and continuous	90 days	NH_4_^+^ salts (instead of expensive NO_3_^–^ salts) can be used to commercially grow *L. indica*	Sachdeva et al. (2018a)
Erlenmeyer flasks (batch) and cylindrical double jacketed PBR	Purging with N_2_, stirring with turbine	250 mL (batch), 2 L (PBR)	60 μE m^–2^ s^–1^ (batch) and 300 ± 50 μE m^–2^ s^–1^ (PBR), radially illuminated	Batch and continuous	7 days	Demonstration of potential of using urea and nitrite salts, as cheaper alternatives to nitrate salts	Sachdeva et al. (2018b)
PBR consisting of two glass cylindrical tubes	External-loop airlift	83 L (55 L illuminated volume)	Varying depending on experiment	Continuous	30 and 50 days	A mathematical model to describe a two compartment system is successfully demonstrated. (“crew” = rats and PBR providing O_2_)	Alemany et al. (2019)
Flat, cylindrical PBR	Hollow fibers	2.6 L	20, 35, and 50 W/m^2^ (≈ 92, 161, 230 μE m^–2^ s^–1^), via LED	Quasi-batch	27 days	No excessive shear stress is applied to the bacteria, the model is applicable	Chapuis et al. (2020)

**TABLE 3:** PBR ground experiments on algae and cyanobacteria, fed by a nitrifying culture.

Organisms	Hardware	Gas exchange	Volume	Light intensity	Mode	Duration	Results	Authors
*Limnospira indica* PCC 8005, fed by *Nitrosomonas europaea* ATCC 19178, *Nitrobacter winogradsky* ATCC 14123	PBR, 2 identical cylinders	External loop airlift	7 L and 77 L	white halogen lamps (20 W), between ≈ 100–400 W/m^2^ (≈ 460–1,840 μE m^–2^ s^–1^)	Continuous	4 years, many different experiments and conditions	The separately operated as well as the interconnected bioreactors were successfully run in a continuous way	Gòdia et al. (2002)
Axenic *Limnospira indica*, fed by 12 different nitrifying inocula	Two membrane bioreactors (nitrifying community), 96-well plate (*L. indica*)	Air pump	8 L (bioreactor), 0.3 mL (*L. indica*) and 0.8 L (*L. indica*)	200 μE m^–2^ s^–1^ (0.3 mL batch of *L. indica*), 160 μE m^–2^ s^–1^ (0.8 L batch of *L. indica*)	Batch and continuous	0.8 L batches of *L. indica*: 10 days, bioreactor up to 180 days (diagram)	*Limnospira indica* grew with high rates on the nitrified urine and yielded a high biomass protein content. *Nitrobacter* spp. became the dominant species in the nitrite oxidizing community	Coppens et al. (2016)
Different microalgae species, fed by commercially available nitrifying activated sludge	Plexiglas, gastight PBR	Airlift	4 L	300 μE m^–2^ s^–1^	Semi-continuous	180 days	The biological oxidation of all nitrogen sources in urine was successful and is a promising treatment for nutrient recovery of waste water	Muys et al. (2018)

The bioreactors developed for space used artificial light (e.g., halogen lamps or LEDs) with varying intensities and different designs of hardware were used, e.g., plate, cylindrical and rectangular bioreactor forms (Tables 1–3). The volumes used are generally in lab to pilot scale and range from 100 mL (Cornet and Dussap, 2009) up to 83 L (Alemany et al., 2019). Batch as well as continuous modes were tested. Also, the durations of the experiments vary widely (a few hours up to 4 years). *Limnospira indica* was the most often used organism in these experiments (Table 2). In many experiments, the gas exchange between the liquid and gaseous phase was accomplished by airlift systems. However, due to minimal gravity in space, this aeration system does not work and has to be replaced by e.g., membrane-aerated hardware (Wagner et al., 2015). Literature research revealed additional PBR research for space applications with other microorganisms. One interesting example is the ModuLES project with the green alga *Chlamydomonas reinhardtii* grown in a membrane-aerated plate-type PBR. Two liters of culture were grown continuously and the system was successfully tested in two parabolic flight campaigns (Wagner et al., 2015).

Additionally, many experiments using higher plants as photosynthetic organisms have been done, but these approaches are not covered in these tables.

### Testing Space PBRs in Space Flight

**FIGURE 1:**
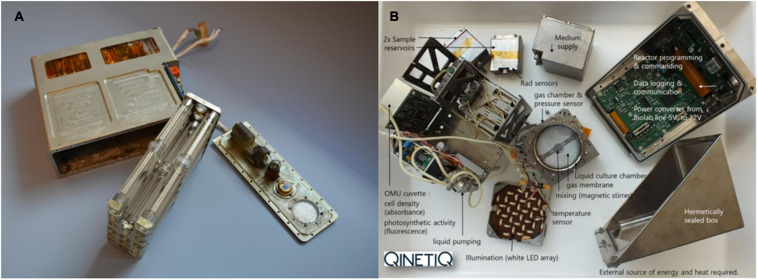
**(A)** *Nostoc* sp./*Euglena gracilis* container of the first space flight experiment associated with MELiSSA (Dubertret et al., 1987), courtesy of ESA. **(B)** *Limnospira indica* hardware from the ArtEMISS-B experiment (Poughon et al., 2020, original source: QINETIQ).

So far, micro-gravity experiments in space only contributed small parts to the development of different life support systems. Space experiments are very expensive, need extensive preparation and the precious crew-time of astronauts (Leys et al., 2004). Nevertheless, some experiments on liquid culturing and photobioreactors have already been performed in space (Table 4). Depending on the scientific question, many different experimental setups were used, i.e., liquid cultivation using different tube and flask designs up to fully assembled bioreactors (Poughon et al., 2020). Most conducted experiments aimed for very fundamental questions like how liquid cultures respond to microgravity and space radiation. The earliest record for algae flown to space dates back to 1960. In this 25 h experiment on Korabl-Sputnik 2, the algae were grown on agar in the dark and in liquid culture under periodic artificial illumination. Since some cells survived the flight and were able to grow and reproduce, it was concluded that algae can perform their basic physiological and photosynthetic functions in orbit (Semenenko and Vladimirova, 1961). More elaborate experiments were conducted in the years afterward, using different photosynthetic organisms on space stations (Mir, ISS) and on free flying return capsules. In the 1960s, 1970s, and 1980s, common organisms were *Chlorella pyrenoidosa*, *Chlorella vulgaris* and *Chlamydomonas reinhardtii* (Niederwieser et al., 2018). In 1987, the cyanobacterium *Nostoc sp.* PCC7524 and a plastid mutant of the eukaryotic alga *Euglena gracilis* flew on board a Long March II Chinese rocket to space. In this experiment, the plastid mutant alga was the O_2_ consumer and CO2 producer and the cyanobacterium produced O_2_ and consumed CO_2_, respectively. It could be shown, that both organisms survived the 4.5 days in space and that some of the cyanobacteria grew under illumination (Dubertret et al., 1987). The used hardware of this early experiment is shown in Figure 1. This experiment is often referred to as the origin of MELiSSA (Lasseur and Mergeay, 2021, accepted for publication). In the later years, there were also some approaches with cyanobacteria like *Nostoc commune* var. *sphaeroides* (Wang et al., 2004), *Anabaena siamensis* (Wang et al., 2006), and *Limnospira indica* (Ilgrande et al., 2019). The latter was used in the Arthrospira-B experiment which was launched to the ISS in 2017 and which was the first approach to allow online measurements of the O_2_ production rate as well as growth rate in space. Additionally, the cultures were kept axenic over the entire duration of over 1 month (Poughon et al., 2020). To date, this experiment is the most sophisticated successful approach to run an instrumented photobioreactor onboard a space station (Figure 1).

**TABLE 4:** PBR space flight experiments with algae and cyanobacteria (∼ last 30 years, only published ones, chronologically listed).

Organism	Vehicle	Hardware	Volume	Light intensity	Mode	Duration	Results	Authors
*Nostoc sp.* PCC7524 and a plastid mutant of *Euglena gracilis*	Long March 2	Dialysis bags that allow for gas exchange	3 mL per culture	0.3 W bulb	1 batch	4.5 days	Fixation of the cells was successful, some of the *Nostoc* cells germinated in the microgravity conditions	Dubertret et al. (1987)
*Chlorella vulgaris* LARG-1	Bion-9 (Cosmos, 2044)	Three-component aquatic system	Unknown	Unknown, but it was illuminated	1 batch	13 days	Microscopy revealed differences in organelle-organization between space and ground samples but there was so significant difference in growth	Popova et al. (1989)
*Nostoc sphaeroides* Kütz	Shenzhou-II	Closed chambers	85 mL	2,200 Lux, 12-h-dark/12-h-light cycle	1 batch	6 days 15 h	A high growth rate was observed for the space samples exposed to microgravity	Wang et al. (2004)
*Anabaena siamensis* FACHB 799	Chinese retrievable satellite	small bioreactor	200 mL	15 μE m^–2^ s^–1^	1 batch	15 days	Growth in space was slower, but after return, the space cultures grew at a higher rate. After a few generations, both cultures grew at the same rate	Wang et al. (2006)
*Euglena gracilis* with *Oreochromis mossambicus* (cichlid fish)	Foton M2	Cylindrical prototype with two connected bioreactors	1.45 L (*E. gracilis*) and 1.26 L (fish)	Via red LEDs (emission peak at 625 nm)	Continuous	15 days	The oxygen production of *E. gracilis* gradually decreased in the first 9 days and increased afterward. Seven of the 35 fish died	Häder et al. (2006)
*Chlorella pyrenoidosa* FACHB 415 and *Bulinus australianus* (snail)	Chinese retrievable satellite and Shenzhou-II	Culture chambers	85 mL algal culture in 120 mL chamber	35 μE m^–2^ s^–1^	1 batch	Satellite: 15 days; spacecraft: 6 days 15 h	Satellite: The algae survived but became a little lower in number, the snails died (probably from CO2 intoxication), spacecraft: the average *Chlorella* concentration decreased	Wang et al. (2008)
*Euglena gracilis* with *Oreochromis mossambicus* (cichlid fish)	Foton M3	Polycarbonate cylinder with adjacent compartments	Unknown	Via three pairs of high-power red LEDs	Continuous	12 days	The oxygen level in the tank decreased a little more than expected. 11 out of 26 fishes survived the flight	Strauch et al. (2008)
*Euglena gracilis* Z	Shenzhou-8	Double culture chamber, separated by biofoil	11 mL	500 μE m^–2^ s^–1^	1 batch	17 days, fixation after 40 min (other samples failed)	First report on microgravity-induced changes at the transcriptional level of an unicellular eukaryotic organism	Nasir et al. (2014), Preu and Braun (2014)
*Limnospira indica* PCC8005	ISS	Cylindrical PBR with flat membrane liquid and gaseous phase	60 mL	35 and 45 μE m^–2^ s^–1^	Batches (14, 6, 8, 6 days)	5 weeks	Generally successful, but some technical difficulties. First dynamic growth of cyanobacteria in space and the gas and biomass model was shown to be applicable	Poughon et al. (2020)

Although several experiments using photobioreactors in space were conducted, many of them were unsuccessful or the data were never reported in publications (Niederwieser et al., 2018). The first experiment with in-flight analysis on *Chlorella sorokiniana* in liquid medium for 30 days failed because the reactor had a leak and exposed the algae to vacuum (Ward et al., 1970). A more recent unsuccessful experiment is the Eu:CROPIS project described in Hauslage et al. (2018). In this life support system experiment, *Euglena gracilis* was used in combination with a tomato plant and different bacteria (*Nitrosomonas*, *Nitrobacter*) to produce O_2_ and edible biomass. Unfortunately, the flight experiment did not result in usable data due to a technical failure. Another example is the PBR@LSR project. In this project, a PBR inoculated with *Chlorella vulgaris* was brought to the ISS in 2019. The proposed experiment time was 6 months, but the experiment had to be terminated after a few weeks due to technical issues (Keppler et al., 2018). Table 4 shows a selection of space flight experiments using photosynthetic unicellular organisms in illuminated test chambers (PBRs) that have been in space over the last 30 years. Since not all of the experiments in space have been successful, not all space experiments were published so that only experiments published in peer reviewed papers that could be used as references are shown.

## Discussion

In this section, we highlight the different challenges in context of PBR for space applications in order to give an overview of knowledge gaps and problems that already occurred or might arise in the future of BLSS research.

### Safety and Reliability – Robustness, Resilience, and Redundancy

The safety and reliability of a life support system is of utmost importance. In order to avert fatal incidents, several back-up facilities and control mechanisms have to be installed and the system has to be monitored consistently. All possible scenarios have to be calculated and evaluated beforehand to avoid failure, because failure can be fatal for the crew (Bartsev et al., 1996). For example, a failure in O_2_ production has to be intercepted by an emergency system before a drop in the O_2_ concentration of the cabin occurs. A high degree of redundancy has to be achieved. Physicochemical emergency back-up systems, plant compartments and different PBRs could be put in parallel that can be uncoupled from each other. And not all bioreactors need to be operated in long duration continuous production, but a regime of alternating batches or operation and downtime of bioreactors could be implemented, if shown to be advantageous for operation, harvesting or maintenance. In addition, reliable mathematical models for the bioreactors are essential to keep all processes predictable (Vernerey et al., 2001). This is a highly strategic point when the recycling efficiencies of different elements and compounds are coupled and intertwined as it is the case for BLSS, such as MELiSSA. In this case, the action on an operational variable has distributed consequences at several points of the recycling system, calling for an intelligent control strategy based on knowledge models taking into account the dynamic exchanges between the different parts of the recycling system. The other important point for life support systems for space is that the buffer tanks generally have a minimal capacity entailing an online control strategy. The criteria for reliability, availability, maintainability and supportability (RAMS) engineering have to be applied in the BLSS research.

### Gas Exchange and O_2_/CO_2_ Balance Between Consumer and Producer

As mentioned, on average, one human needs ∼0.82 kg of O_2_ and produces ∼1.04 kg of CO_2_ per day. Depending on the activity level, the ratio of exhaled CO_2_ to consumed O_2_, i.e., the respiratory coefficient, can vary (Anderson et al., 2018). On the other hand, the O_2_ production of algae and cyanobacteria can be characterized by a photosynthetic coefficient, describing the ratio of produced moles of O_2_ per consumed moles of CO_2_. This ratio is dependent on the organism (and its biochemical composition) and the nutrient substrate (e.g., the nitrogen source). The stoichiometric eqs. 3, 4 [simplified from Cornet et al. (1998)] show this dependence for *Limnospira indica* on the examples ammonium (NH_3_) and nitrate (NO_3_^–^ or here: HNO_3_) as nitrogen sources. Solving the stoichiometric equations reveals that the photosynthetic coefficient for ammonium is ∼1.0 and for nitrate ∼1.4.

(3) CO+20.528HO2+0.173NH+3nhν→CHO1.575N0.4590.173+1.034O2

(4) CO+20.701HO2+0.173HNO+3nhν→CHO1.575N0.4590.173+1.381O2

Importantly, it must be outlined that photosynthetic growth stoichiometry has no degree of freedom when the composition of nitrogen source (or its degree of reduction) is fixed so that the photosynthetic coefficient is only depending and linked to the culture conditions. The number of photons required to fix 1 mol of carbon is depending on the culture conditions. Away from photo inhibition conditions, a typical value is *n* = 20 mol photons per mol of carbon fixed (Cornet and Dussap, 2009; Poulet et al., 2020).

In order to avoid an imbalance in gas composition, a system has to be developed to combine the respiratory quotient of the crew members and the photosynthetic coefficient of the microalgae. In some experiments, successes were achieved (see section “Photosynthetic Microorganisms as Catalysers for Air Revitalization in Space”). However, gas exchange in space is much more complex, due to the lower or lack of gravity. There is still very little information about the gas, water and solute transport in microgravity in living organisms. Moreover, microgravity conditions strongly modify the environment of the chemical and biochemical processes, e.g., implying lack of sedimentation and impaired gas and liquid phase separation. Consequently, transport is limited to diffusion causing an increase of boundary layer thickness and therefore a significant decrease of mass and heat transfer coefficients. This can cause problems with pumping and the mineral availability for the cultures and has to be elucidated more thoroughly (Klaus et al., 1997).

### *In situ* Resource Utilization and Light as an Energy Source

Another challenge is the complete closure of a BLSS. So far, no loop has an efficiency of 100%, which means that all tested life support systems still rely on external addition of different substances like carbonate or trace elements, etc. For example, the 105 days long Lunar Palace 1 experiment (plants, insects, and three crew members) reached a full oxygen and water recycling but only 20.5% nitrogen recovery from urine and 55% of the food was regenerated. In this approach, physico-chemical and biological processes were combined (Fu et al., 2016). Some substances are either difficult to find in the space environment or it is very costly and time consuming to convert them into a usable form. Therefore, space habitats for humans have to be fully functional under the specific conditions and have to rely on the materials available around and only a small amount of material brought from Earth. For example, lunar regolith, mars soil and CO_2_ in the Martian atmosphere are promising substances to be used for *in situ* resource utilization (ISRU) (Montague et al., 2012; Muscatello and Santiago-Maldonado, 2012).

The usage of photoautotrophic organisms helps to overcome parts of the material problems because their main energy source is light. But so far, only experiments using artificial light (e.g., halogen lamps or LEDs) have been flown (Table 4) which means that the naturally available solar energy is not used directly so far. One of the main reasons is that the natural light intensities and spectral energy distributions available in space are not compatible with the needs of the photosynthetic organisms. The intensity of sun light depends on the distance from the sun and the irradiation spectrum in deep space consists of a different wavelength composition than the irradiation we experience on ground due to absorption of light in the Earth’s atmosphere (Cockell and Horneck, 2001). Besides that, the ISS, Moon and Mars surface are eclipsed for 50% of the time and the day and night cycles, e.g., on the moon are very different from Earth. For example, one lunar night is as long as 18 Earth days (Alvarado et al., 2021; Xie et al., submitted)^1^. Also, the intensity of natural light sources is much more difficult to be controlled than artificial light sources. Therefore, approaches where solar power is used to store energy in batteries as fuel for LEDs with suitable light characteristics, that can be used in PBRs and greenhouses, are desired.

### Scaling Up

So far, the research on the PBR part of the BLSS is the most sophisticated area. But also in the PBR research, most experiments were done in lab up to pilot scale (100 mL up to 83 L, Tables 1–4) and these volumes were not sufficient to provide 100% of the O_2_ need of a crew member (Javanmardian and Palsson, 1992; Alemany et al., 2019). So even if a small lab scale bioreactor is successful, scaling-up procedures have to follow to achieve the needed production rates for a BLSS, which vary strongly depending on the used organisms (Vernerey et al., 2001). In order to develop a reliable system, the PBR needs to represent a well-balanced combination of a relatively small, but sufficient volume and high productivity via usage of proper illumination in high cell density cultures.

### Connecting Multiple Bioreactors and Closing the Loop

Only a few experiments involve bioreactors that are connected to other life support compartments like the crew or a waste recycling compartment. Consequently, many challenges remain in this research area and the connection between the different systems has to be elucidated more. Additional experiments on the nitrifying community and the other parts of the waste treatment (e.g., thermophilic anaerobic bacteria to produce volatile fatty acids out of waste) have to be conducted in space and further developed on ground (Lasseur et al., 2010). Some unidentified problems might arise in connected bioreactors. Cross-contamination and cross-talking of the organisms by quorum sensing molecules between the different compartments might disturb the system on a long-term scale (Mastroleo et al., 2013). However, also in axenic bioreactors, there is missing knowledge about cell-cell communication and biofilm formation in space.

### Long Duration Cultivation in Engineered Bioreactors and Space Conditions

The following question has to be answered: does a long-term cultivation in engineered bioreactors under space conditions have an effect on the microorganisms? A predictable and stable growth rate of the culture is essential for the performance of a PBR. Early stress signals have to be monitored to avoid culture failure in space and adequate countermeasures have to be developed. For this, low-dose prolonged irradiation and (simulated) microgravity experiments are needed. The genetic stability over multiple generations has to be addressed, e.g., the mutation rates, differences in gene expression and epigenetic effects like changes in DNA methylation patterns. So far, not enough experiments on long term conduction of photobioreactors were done, to answer that question.

The effect of cosmic irradiation and reduced gravity on the oxygen production rate and nutritive value of the photosynthetic microorganisms has to be investigated. In plant experiments, it was reported that low doses of ionizing irradiation can cause an increase in growth rate (Sax, 1963; Upton, 2001), but for algae and cyanobacteria very limited data are available so far. Planel et al. (1987) presented a hormesis effect for the cyanobacterium *Synechococcus lividus* when irradiated with 1.49 mGy per year. If proven for BLSS relevant organisms, such hormesis effects might even be useful for the BLSS productivity.

Most space experiments with living organisms have only been done in LEO so far. There are very few reports of microbial experiments that went out of LEO (Horneck et al., 2010). One of the rare examples is the Chinese lunar chang’e 4 lander that brought organisms (plants, yeast and fruit fly eggs) farther into Space (Xie et al., submitted, see text footnote 1). Therefore, future investigations need to be beyond LEO, and e.g., in Moon orbit or on the Moon surface. Also, insightful investigations remain to be systematically done in order to detail and understand the mechanisms of interaction of zero-gravity conditions and different levels of intracellular organization and metabolic regulation. By example, it is well-known that gravitropism is an important phenomenon for higher plants growth and roots development. Similar effects are likely to occur even in simpler prokaryotic organisms linked to dissolved gas exchanges (namely O_2_ and CO_2_) between intracellular level and the culture environment.

### Remote Commanding, Monitoring, Reporting, and Data Exploitation

During literature research, it became apparent, that the published data often do not include all needed information. Especially the light intensity inside the culture, temperature and pH data are often missing.

Furthermore, the data of space experiments are deficient and often difficult to compare. For example, different sizes for different missions were used (Table 4) and the space experiments were mainly to investigate on exposure, survival and simplified processes. Also, only very few experiments with on-board monitoring can be found in the literature [e.g., ArtEMISS-B described in Poughon et al. (2020)]. Real-time bioprocess monitoring has to be achieved to obtain reproducible and reliable results.

## Conclusion

Bioregenerative life support systems are complex networks of biological and physicochemical transformations, including bioreactors, whose functioning are influenced by space environment like reduced gravity levels and increased doses of ionizing radiation. As a consequence, several biological and physical key bioreactor processes have to be controlled and adapted to these altered conditions by, for example, the usage of membrane-aerated PBRs. In practice, recent flown experiments have shown the challenge linked to the deployment of a successful PBR in space. Further knowledge is therefore needed to improve the necessary success rate that will allow continuous operation at a larger scale.

In general, more long-term continuous experiments should be conducted and all important parameters (temperature, gas exchange rates, light intensity, concentration of nutrients and biomass production) have to be monitored online, allowing remote bioreactor control from Earth, to reduce the dependence on the crew. Even though many promising experiments on photobioreactors for space applications were conducted, the development of a sufficient life support system still depends on an interconnected, continuously running loop system with a maximum closure.

## Author Contributions

JF and NL performed the literature research and writing of the first draft. FM, C-GD, and NL contributed to reviewing and editing. All authors contributed to the article and approved the submitted version.

## Conflict of Interest

The authors declare that the research was conducted in the absence of any commercial or financial relationships that could be construed as a potential conflict of interest.

## Footnotes

1 Xie, G., Zhang, Y., Yang, J., Yu, D., Ren, M., Qiu, D., et al. (submitted). *Adaptationto real 1/6 g Moon Gravity Contributes To Plant Development And Expeditious Acclimation To Super-Freezing*. Chongqing, Research Square. doi: 10.1007/s00204-016-1744-5
